# Bioinformatics analysis identifies heparan sulfate proteoglycans acting as different progress subtypes of biliary atresia

**DOI:** 10.3389/fped.2023.1065521

**Published:** 2023-02-02

**Authors:** Zequan Ding, Wenyu Song, Wei Zhu, Hua Xie, Zhongxian Zhu, Weibing Tang

**Affiliations:** ^1^ Department of Pediatric Surgery, Children's Hospital of Nanjing Medical University, Nanjing, China; ^2^ Department of Cardiovascular Surgery, Zhongshan Hospital, Fudan University, Shanghai, China

**Keywords:** heparan sulfate proteoglycan, biliary atresia, liver fibrosis, ductular reaction, immunocytes infiltration, molecular classification

## Abstract

**Background:**

Biliary atresia (BA) is a life-threatening disorder, which is characterized by the obliteration of biliary tracts. Heparan sulfate proteoglycans (HSPGs) are important regulators in liver diseases. Whether HSPGs participate in the development of BA is poorly understood.

**Methods:**

RNA-seq dataset GSE122340, including 171 BA and 7 normal liver tissue, was integrated for bioinformatic analysis. R function “wilcox.test” was used to compare HSPGs expression levels, and “cor.test” was used to evaluate the correlation analysis. MCPcounter was used to assess the abundance of immunocytes. Molecular subtypes of BA were clustered *via* NMF clustering and LASSO regression was applied to screen hub HSPGs genes in BA clusters. RT-PCR analysis was used to assess the expression of hub HSPGs in BA liver. Immunohistochemical staining and immunofluorescence assay were used to evaluated the location and expression of hub HSPGs in BA liver tissue.

**Results:**

Majority of HSPGs was up-regulated in BA and correlated with liver fibrosis and ductular reaction markers. The abundance of immunocytes was higher in BA and associated with HSPGs. Based on the expression of HSPGs, BA patients were classified into 3 subtypes (C1, C2, and C3). Pathway enrichment analysis revealed C1 subtype had severe liver injury with SDC4 identified as the hub gene, while C3 subtype presented relatively normal liver condition with GPC3 identified as the hub gene. RT-PCR analysis demonstrated the expression levels of 2 hub genes in BA liver tissue with different jaundice clearance standards. Immunohistochemical staining and immunofluorescence assay showed that SDC4 was mostly expressed in ductular reaction area, while GPC3 was mostly expressed in hepatocytes.

**Conclusion:**

Majority of HSPGs are aberrant expressed in BA. The subtype hub gene SDC4 and GPC3 might be used as a potential indicator for different types of prognosis.

## Introduction

Biliary atresia (BA) is the most common etiology of neonatal cholestasis with poor prognosis, which is characterized by the obliteration of both extrahepatic and intrahepatic biliary tree (1). Currently, Kasai portoenterostomy is recommended as a primary treatment to restore bile drainage. However, despite performing Kasai surgery, nearly half of them could not survive with native liver in long-term and liver transplantation will eventually be required (2).

Several histological features are thought to be associated with native liver survival. Firstly, liver fibrosis (marker as ACTA2, COL1A1, COL1A2, COL3A1), reflected by proliferation of myofibroblasts and collagen deposition, is associated with decreased native liver survival mostly (3). Due to the lack of effective treatment of progressive fibrosis, most BA patients eventually require a liver transplant. Secondly, ductular reaction (marker as EPCAM, KRT7, KRT19), defined as the proliferation of bile ducts, contributes to a faster fibrotic manner (4). Moreover, reactive ductular cells, which is the epithelial component of ductular reaction, tend to cross-talk with immunocytes infiltration in the microenvironment (5). Lastly, the disordered immune system is central among the factors linked to the pathologic changes in BA (6). The infiltration of immunocytes and over-expression of the cytokines and chemokines were observed in BA liver. The immune profiling revealed the dysregulation of the immune system and proved the evidence for immune-cell-modifying therapies (7).

Heparan sulfate proteoglycans (HSPGs) are macromolecules, composed of several heparan sulfate glycosaminoglycan chains attached to a core protein (8). Depending on the core protein, HSPGs are classified into three types including syndecans (SDC1–4), glypicans (GPC1–6) and extracellular HPSGs (AGRN, HSPG2 and COL18A1). Previous studies have identified HSPGs as crucial regulators and diagnostic markers in liver diseases. For example, SDC1 has been reported to be positively correlated with the severity of liver fibrosis in several chronic liver diseases (9). In chronic cholestasis, SDC1 and SDC4 have been demonstrated to be associated with ductular reaction (10). Moreover, HSPGs have the abilities to bind to more than 400 bioactive protein ligands and regulate the phenotype and behavior of immune cells (11). Although several studies have reported the anomalous expression of some specific HSPGs in BA, the landscape of HSPG family members has not been comprehensively described (12–14).

In this study, we performed a bioinformatic analysis to evaluate the expression level of HSPGs in BA. We found that high expression of HSPGs was associated with the activation of liver fibrosis, ductular reaction and immune disorder. In addition, a novel molecular classification of BA based on the expression of HSPGs has been established.

## Methods and materials

### RNA sequencing (RNA-seq) data acquisition

The RNA-seq dataset GSE122340 was downloaded from the GEO database (https://www.ncbi.nlm.nih.gov/geo/) (15). The downloaded RNA-seq data was comprised of liver tissue from 171 BA patients and 7 normal controls (NC). The BA data was grouped as publisher previously reported, including the discovery cohort (*n* = 121) and the validation cohort (*n* = 50). Detailed dataset were available in Supplementary Table S1.

### Protein-protein interaction (PPI) network

The HSPGs and genes related to liver fibrosis and ductular reaction were uploaded to the STRING database (https://www.string-db.org/) to analyze protein functional interaction relationships.

### Principal component analysis (PCA)

The heterogeneity of BA and NC tissues based on HSPGs expression levels was distinguished by PCA analysis. Results were visualized using R package “scatterplot3d” (version 0.3-42).

### Evaluation of immunocytes distribution

Immunocytes abundance of BA and NC liver transcriptomic data was calculated by Microenvironment Cell Population-counter (MCPcounter) (16). Using R package “MCPcounter” (version 1.2.0), the abundance of 10 kinds of immunocytes, including T cells, CD8 T cells, Cytotoxic lymphocytes, NK cells, B lineage, Monocytic lineage, Myeloid dendritic cells, Neutrophils, Endothelial cells and Fibroblasts, was estimated. Results were visualized using the R package “ggplot2” (version 3.3.5).

### Differential analysis

The difference of the expression levels of each HSPGs, fibrosis and ductular reaction markers and the abundance of immunocytes between BA and NC were analysed using the R function “wilcox.test”. The results were visualized using the R package “ggplot2” (version 3.3.5).

### Identification of BA subtypes

A non-negative matrix factorization (NMF) clustering was used by R package “NMF” (version 0.23.0) to identify BA subgroup based on HSPGs (17). The optimal value of k was obtained when the magnitude of the cophenetic correlation coefficient began to fall drastically in the discovery cohort. R function “consensusmap” was used to plot the heatmap of clustering with the value k from 2 to 10.

### Pathway enrichment analysis

In this study, Gene ontology (GO) enrichment and Kyoto Encyclopedia of Genes and Genomes (KEGG) pathway analysis of the differentially expressed genes (DEGs) were carried out using R package “clusterProfiler” (version 4.0.5) (18, 19). The visualization was handled *via* the R package “ggpubr” (version 0.4.0) and “GOplot” (version 1.0.2).

### Gene set enrichment analysis (GSEA)

GSEA was conduucted using GSEA software (version 3.0), which was downloaded from http://software.broadinstitute.org/gsea/index.jsp. For pathway enrichment analysis, the c2.cp.kegg.v7.4.symbols.gmt was downloaded from the Molecular Signatures Database (http://www.gseamsigdb.org/gsea/downloads.jsp). FDR < 0.25 was considered statistically significant.

### Screening of hub genes

Machine learning algorithm least absolute shrinkage and selection operator (LASSO) regression was applied to screen hub HSPGs genes (20). R package “glmnet” (version 4.1-3) was used to conduct LASSO regression.

### Correlation analysis

Correlation analyses were conducted using Spearman's method *via* the R function “cor.test”. The results were plotted using the R package “corrplot” (version 0.90), “circlize” (version 0.4.15) circlize and “ggplot2” (version 3.3.5).

### Patients’ information

The study enrolled patients with BA (*n* = 39) first treated at the Children's Hospital of Nanjing Medical University, Nanjing, China. The diagnosis of BA was based on intraoperative biliary tract exploration combined with histological features of liver biopsies. This study was approved by the Ethics Committee of the Children's Hospital of Nanjing Medical University and all participants’ parents signed informed consents.

Clinical characteristics were assessed for all patients, including sex, the age when underwent the Kasai portoenterostomy, level of direct bilirubin (DBil) before surgery and DBil level 3 months after surgery. Based on jaundice clearance (DBil level) at 3 months post-Kasai surgery, all patients were divided into 2 groups, jaundice group (DBil > 34.2 μmol/L) and jaundice-free group (DBil ≤ 34.2 μmol/L).

### Quantitative RT-PCR

Total RNA was obtained from biopsy liver tissue using TRIzol reagent as described by the manufacturer (Invitrogen Life Technologies Co, USA). Quantitative RT-PCR was performed to determine the expression levels of SCD4 and GPC3. For mRNA detection, total RNAs (500 ng) were reverse transcribed using the reverse transcription kit (Takara, Tokyo, Japan) under 37°C for 15 min and 85°C for 30 s. RT-PCR was performed on the ABI Prism 7900HT (Applied Biosystems, USA). β-actin was used as an endogenous control. Forward and reverse primer's sequences of SCD4 and GPC3 were as follows: SDC4 former primer: GGACCTCCTAGAAGGCCGATA, reverse primer: AGGGCCGATCATGGAGTCTT; GPC3 former primer: CCTTTGAAATTGTTGTTCGCCA, reverse primer: CCTGGGTTCATTAGCTGGGTA. PCR was performed for 5 s at 95°C and for 30 s at 60°C for 40 cycles.

### Immunohistochemical and immunofluorescence assay

The specimens were fixed with 4% paraformaldehyde (PFA) and embedded in paraffin after tissue processing. After deparaffinization, rehydration, and antigen retrieval, 2-µm-thick sections were blocked with 10% normal donkey serum for 1 h at room temperature and stained with primary antibodies. The following primary antibodies were used in immunohistochemical staining: anti-SDC4 (1:500; Proteintech, Wuhan, China) and anti-GPC3 (1:500; Proteintech, Wuhan, China). Biotinylated anti-rabbit (Beyond, Shanghai, China) antibody was used as secondary antibodies according to the manufacturer's protocol. 3,3N-Diaminobenzidine Tertrahydrochloride (Beyond, Shanghai, China) was used as a chromogen according to the manufacturer's protocol. For double-immunofluorescence analysis, sections were pretreated in the same way as for IHC. The following primary antibodies were used: anti-SDC4 (1:50; mouse; Santa Cruz Biotechnology) and anti-KRT19 (1:500; rabbit; Abcam); anti-GPC3 (1:500; rabbit; Abcam) and anti-ACTA2 (1:500; mouse; Abcam). All slides were incubated with primary antibody overnight, which was diluted with 10% normal donkey serum after 1-h serum blocking. After DAPI (4’6-diamidino-2-phenylindole) staining, images were acquired using an Olympus BX51 microscope.

### Statistical analysis

Continuous variables were presented as mean ± standard deviations (SD), and qualitative variable data were presented as a percentage with a 95% confidence interval. The Student's *t*-test was used for the comparison of measurement data between the 2 groups. For comparisons of enumeration data, Fisher's exact test was used. *p*-value < 0.05 was considered statistically significant. All analyses were executed by GraphPad Prism 8 software (GraphPad Software Inc., California, USA).

## Results

### Aberrant expression of HSPGs in BA

**Figure 1:**
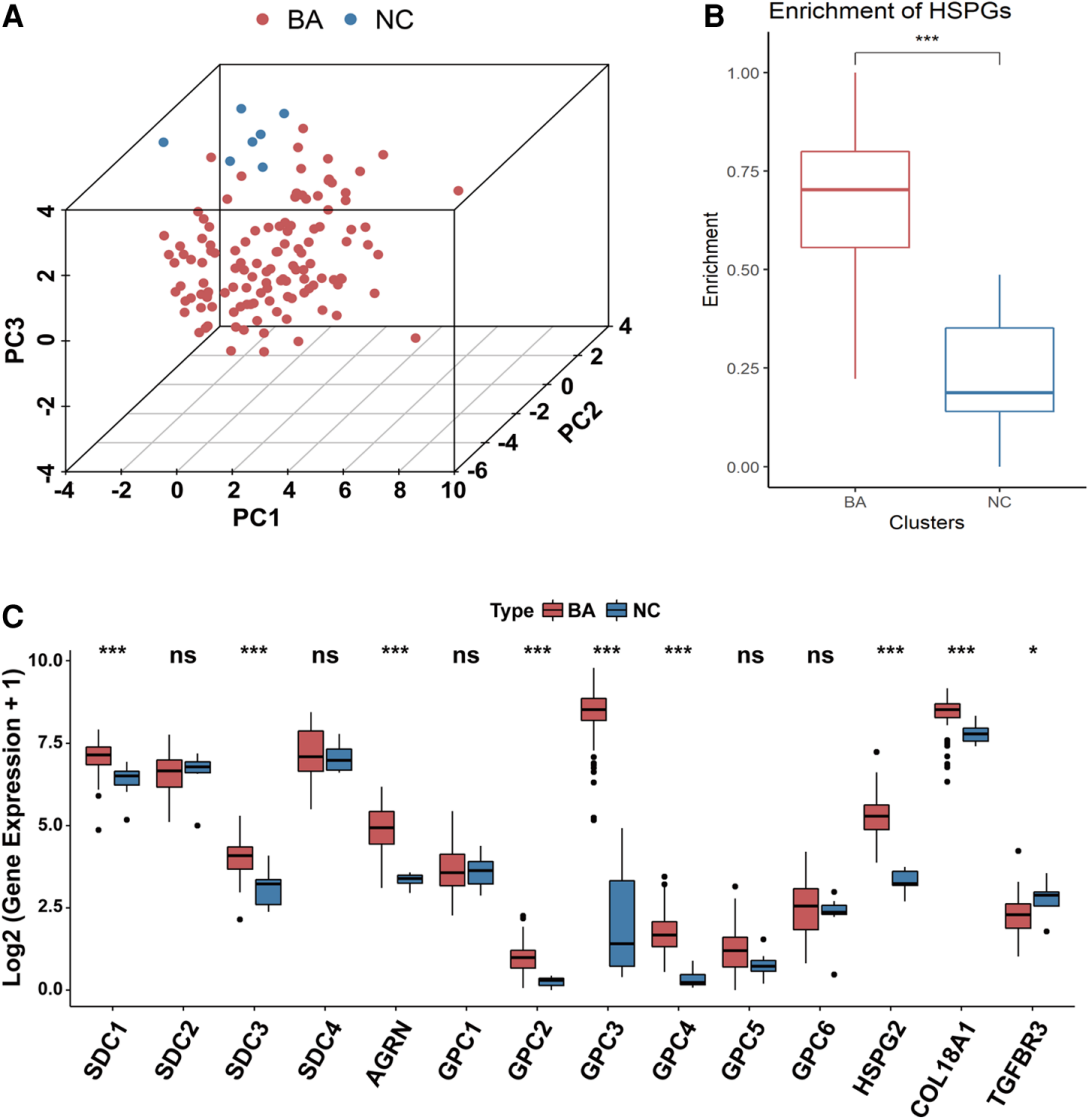
Aberrant expression of HSPGs in BA. (**A**) The dot plot of PCA of BA and NC tissues based on HSPGs expression levels. (**B**) The enrichment score of HSPGs in BA and NC livers. (**C**) The expression levels of HSPGs in BA and NC livers. HSPG, heparan sulfate proteoglycan; PCA, principal component analysis; BA, biliary atresia; NC, normal control. ns, not significant, *p < 0.05, **p < 0.01, ***p < 0.001.

PCA analysis showed the heterogeneity of BA and NC tissues based on HSPGs expression levels (Figure 1A). The enrichment score of HSPGs was higher in BA using ssGSEA analysis (Figure 1B). SDC1, SDC3, AGRN, GPC2, GPC3, GPC4, HSPG2, COL18A1 and TGFBR3 were significantly upregulated in BA (Figure 1C and Table 1).

**Table 1:** Logfc and *p*-value of heparan sulfate proteoglycans.

Gene	logFC	*p*-value
SDC1	0.723032	0.000719
SDC2	−0.00687	0.974685
SDC3	1.03583	0.001359
SDC4	0.276071	0.367056
AGRN	1.747248	1.78 × 10^−5^
GPC1	0.190459	0.588252
GPC2	2.404978	0.000691
GPC3	5.384131	2.05 × 10^−8^
GPC4	3.143244	0.000554
GPC5	0.983661	0.110504
GPC6	0.314266	0.391331
HSPG2	2.12975	3.16 × 10^−5^
COL18A1	0.681362	0.000376
TGFBR3	−0.58185	0.01584

LogFC, log fold change.

### The association between the expression of HSPGs and liver fibrosis and ductular reaction in BA

**Figure 2:**
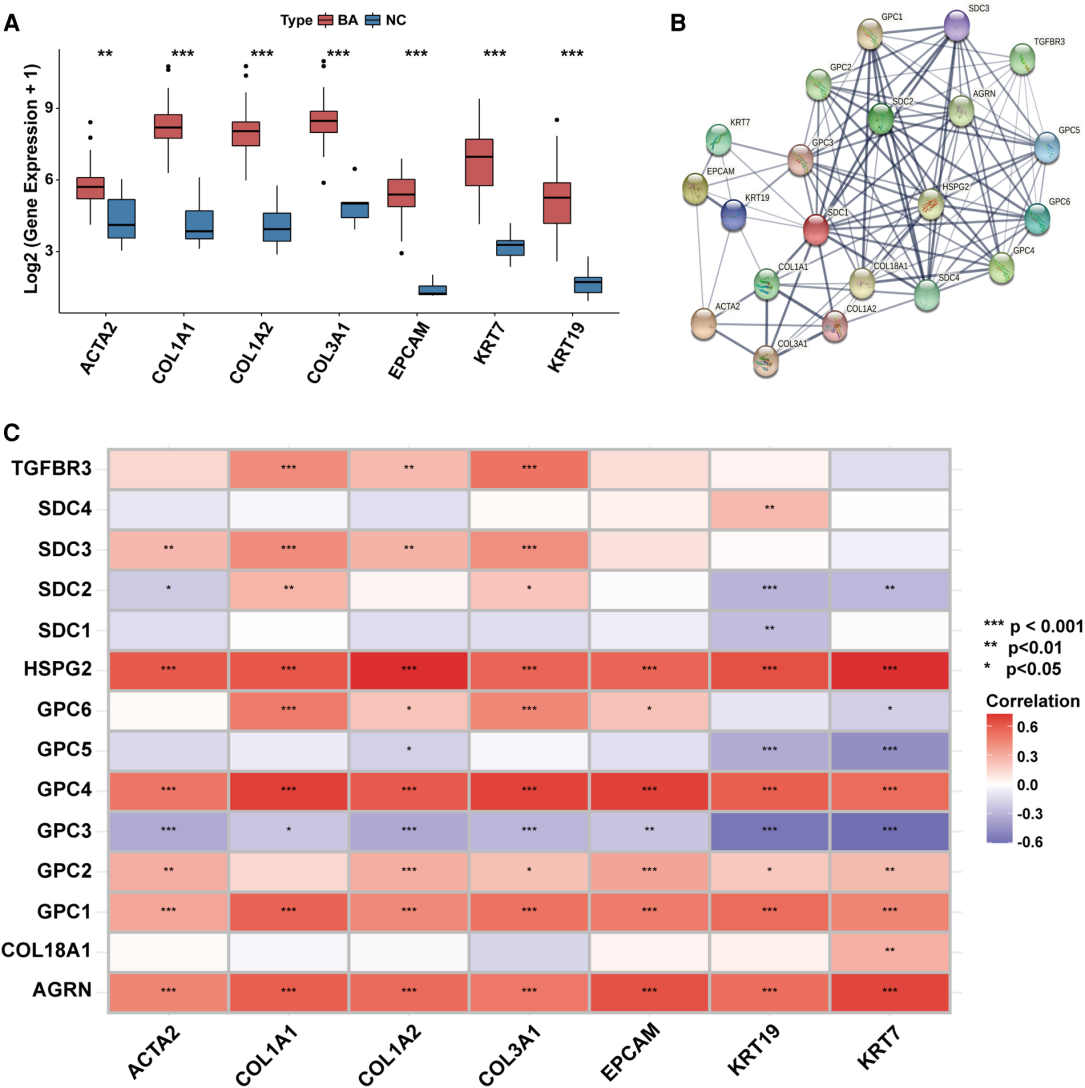
The association between the expression of HSPGs and liver fibrosis and ductular reaction in BA. (**A**) Liver fibrosis and ductular reaction indexes expressions in BA and NC livers. (**B**) PPI network of HSPGs, liver fibrosis and ductular reaction indexes. Each node represents a type of protein, and edges connect the interacted index. (**C**) Correlation between HSPGs, liver fibrosis and ductular reaction indexes in BA. Red represents higher correlation and blue represents lower correlation, respectively. HSPG, heparan sulfate proteoglycan; BA, biliary atresia; NC, normal control; PPI, protein-protein interaction. ns, not significant, *p < 0.05, **p < 0.01, ***p < 0.001.

Transcriptional levels of liver fibrosis markers (ACTA2, COL1A1, COL1A2, COL3A1) and ductular reaction markers (EPCAM, KRT7, KRT19) were significantly upregulated in BA (Figure 2A).

HSPGs were shown in a PPI network with liver fibrosis and ductular reaction markers (Figure 2B). The expressions of AGRN, GPC1, GPC2, GPC4, HSPG2 were positively correlated with fibrosis and ductular reaction markers in BA, while SDC3 was only with fibrosis markers (Figure 2C). Although GPC3 expression was higher in BA than NC, it was negatively correlated with markers of these 2 features. The similar results were confirmed in the validation cohort (Supplementary Figure S1).

### The association between the expression of HSPGs and immmunocyte infiltration in BA

**Figure 3:**
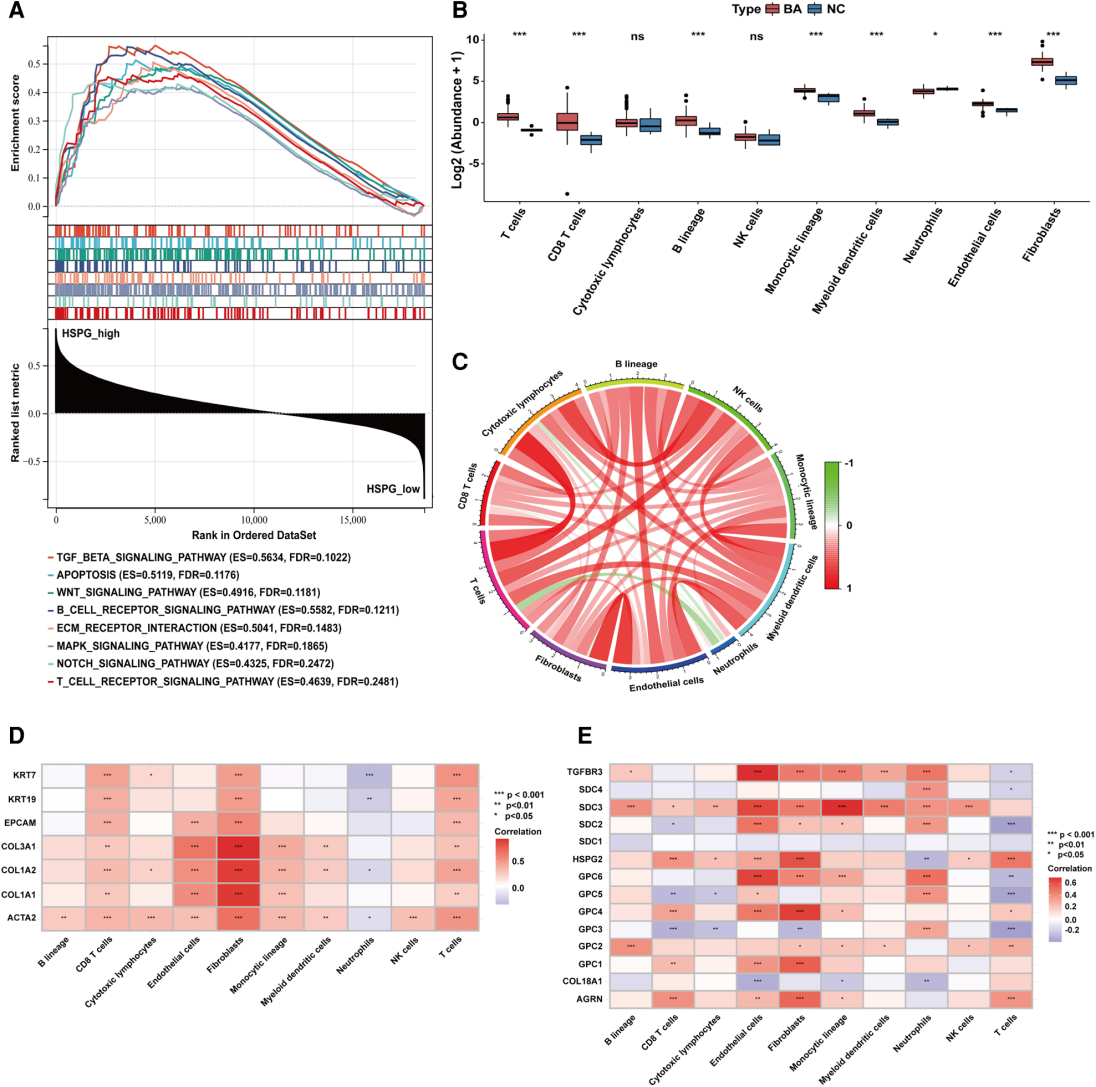
Immunocytes infiltration in BA and NC livers. (**A**) GSEA of BA patients classified in 2 groups based on HSPGs ssGSEA enrichment score. (**B**) The composition and abundance of immunocytes calculated by MCPcounter. (**C**) Correlation between immunocytes abundance in BA. (**D**) Correlation between immunocytes abundance, liver fibrosis and ductular reaction markers in BA. Red represents higher correlation and blue represents lower correlation, respectively. (**E**) Correlation between immunocytes abundance and HSPGs in BA. BA, biliary atresia; NC, normal control; GSEA, gene set enrichment analysis; HSPG, heparan sulfate proteoglycan; MCPcounter, microenvironment cell population-counter. *p < 0.05, **p < 0.01, ***p < 0.001.

Based on the median of HSPG ssGSEA enrichment score, 121 BA patients in the dataset were divided into 2 group, HSPG_high (*n* = 61) and HSPG_low (*n* = 60). Via GSEA, several KEGG pathways associated with immune reaction were ranked in HSPG_high group, such as B_CELL_RECEPTOR_SIGNALING_PATHWAY, T_CELL_RECEPTOR_SIGNALING_PATHWAY, ECM_ RECEPTOR_INTERACTION and some singaling pathways (Figure 3A). The GSEA results indicated that HSPGs, reported as regulators of immune activation, might participate in the immunocyte infiltration in BA.

MCPcounter was applied to estimate the relative abundance of immunocytes in BA and NC microenvironment. The abundance of 10 types of immunocytes were shown in Figure 3B, including T cells, CD8 T cells, cytotoxic lymphocytes, B lineage, NK cells, monocytic lineage, myeloid dendritic cells, neutrophils, endothelial cells and fibroblasts. The abundance of T cells, CD8 T cells, B lineage, monocytic lineage, myeloid dendritic cells, endothelial cells and fibroblasts were upregulated in BA, while neutrophils was downregulated. The correlation analysis between immunocytes in BA patients showed the abundance of neutrophils was negatively correlated with some of other immunocytes, such as T cells, CD8 T cells, cytotoxic lymphocytes and fibroblasts (Figure 3C). Figure 3D showed the abundance of most immunocytes were positively correlated with the expression levels of fibrosis and ductular reaction markers in BA, while neutrophils was negatively correlated with these markers. Figure 3E showed that HSPGs were widely correlated with immunocytes in BA patients. Specifically, GPC3 was the only HSPG negatively correlated with the abundance of fibroblasts.

### Identification of BA subtypes based on HSPGs

**Figure 4:**
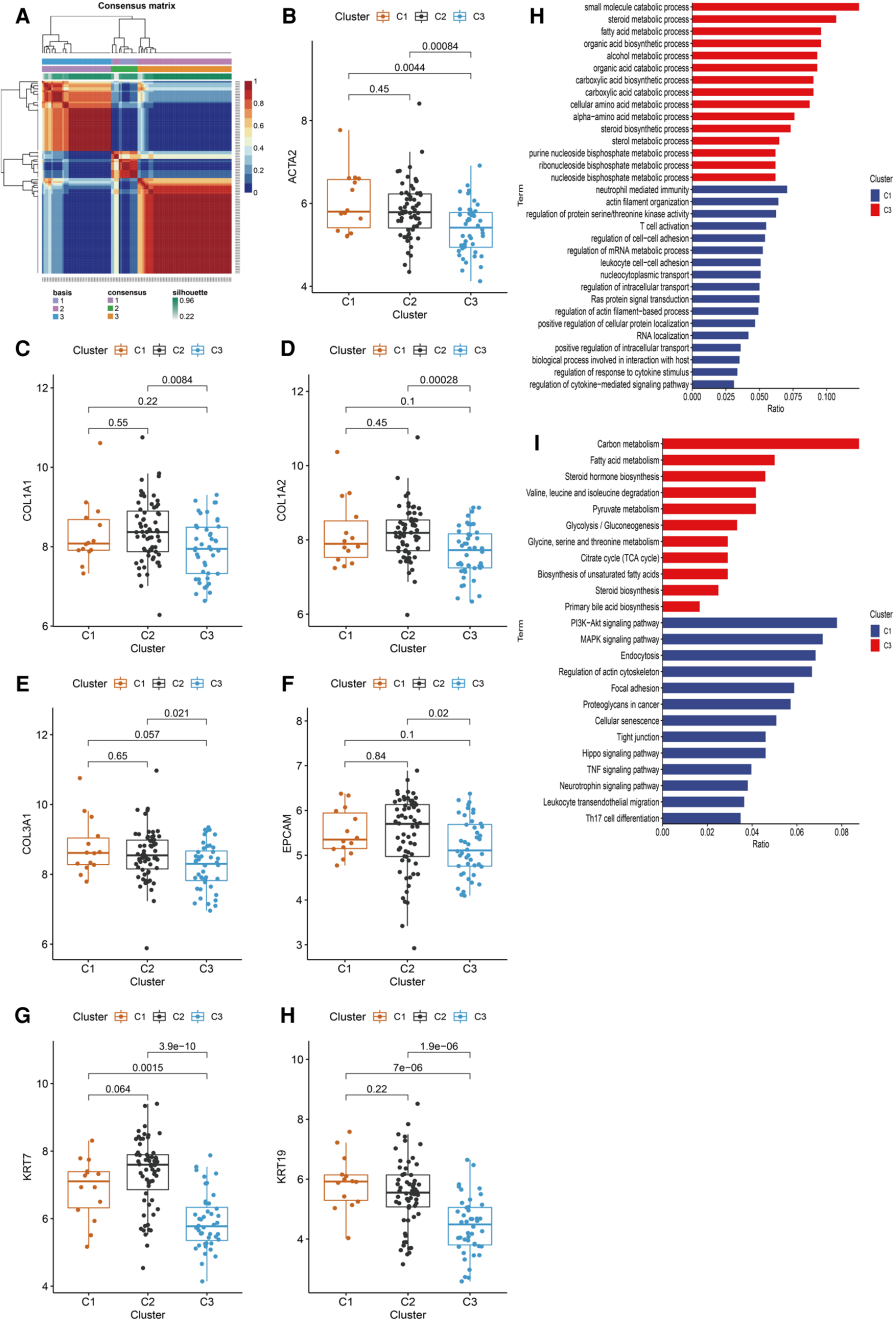
Identification of BA subtypes based on HSPGs expression levels. (**A**) NMF clustering heatmap when *k* = 3. (**B–H**) The expression of ACTA2, COL1A1, COL1A2, COL3A1, EPCAM, KRT7 and KRT19 among three identified subtypes. (**I**) GO enrichment analysis of C1 subtype and C3 subtype. (**J**) KEGG pathway analysis of C1 subtype and C3 subtype. BA, biliary atresia; HSPG, heparan sulfate proteoglycan; NMF, non-negative matrix factorization; GO, gene ontology; KEGG; kyoto encyclopedia of genes and genomes.

HSPGs were then applied to identify molecular subtypes using NMF consensus clustering. NMF heatmap and cophenetic correlation coefficients were shown in Supplementary Figure S2, S3. Optimal number of clusters were chosen as 3 for further analyses (Figure 4A). Then 121 BA patients in discovery cohort were divided into 3 clusters including C1 (*n* = 14), C2 (*n* = 63) and C3 (*n* = 44). Fibrosis and ductular reaction markers in C3 were lower than other clusters, indicating that patients in C3 might have less serious liver injury (Figure 4B–H). SDC2, GPC3 and GPC5 were significantly higher in C3 (Supplementary Figure S4A–N). The abundance of most immunocytes were lower in C3, such as T cells, cytotoxic lymphocytes, fibroblasts (Supplementary Figure S5A–J). GO enrichment analysis revealed that differential expressed genes of C1 were mainly enriched in immune response, cytokine response, cell adhesion, however, metabolic processes was mainly enriched in C3 (Figure 4I). Consistent with GO analyses, KEGG identified enrichment of immune related pathways in C1, such as PI3K-Akt, MAPK, Hippo, TNF signaling pathways, while metabolic pathways were enriched in C3 (Figure 4J). Interestingly, C3 subtype in BA seemed to exhibit relatively modest liver injury, suggesting that C3 patients might have better prognosis.

### Screening of hub HSPGs of C1 and C3 subtype

**Figure 5:**
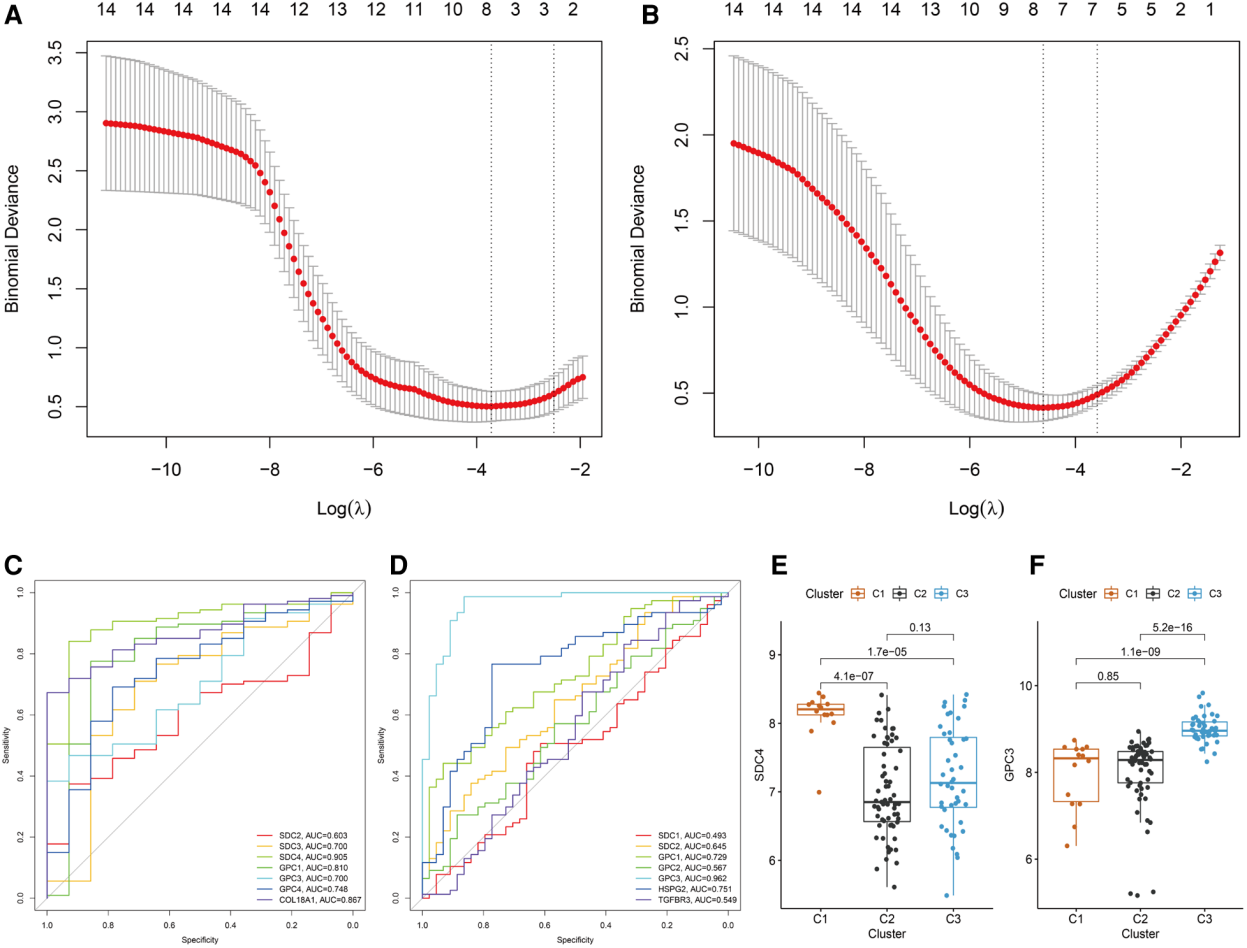
Screening hub HSPGs in C1 subtype and C3 subtype. LASSO regression of screening HSPGs in (**A**) C1 subtype and (**B**) C3 subtype. ROC curve of selected HSPGs in (**C**) C1 subtype and (**D**) C3 subtype. (**E**) The expression levels of SDC4 in 3 subtypes. (**F**) The expression levels of GPC3 in 3 subtypes. HSPG, heparan sulfate proteoglycan; LASSO, least absolute shrinkage and selection operator; ROC, receiver operating characteristic.

Heterogeneity of BA patients was observed based on the expression of HSPGs. HSPGs were screened *via* LASSO regression for the hub HSPGs of C1 and C3 subtype. For C1, SDC2, SDC3, SDC4, GPC1, GPC3, GPC4 and COL18A1 were calculated by LASSO (Figure 5A). For C3, SDC1, SDC2, GPC1, GPC2, GPC3, HSPG2 and COL18A1 were the selected genes (Figure 5B). The ROC curve of these genes showed that SDC4 was the suitable HSPGs for identifying C1 subgroup (AUC = 0.905) (Figure 5C). GPC3 was the suitable HSPGs for C3 subgroup (AUC = 0.962) (Figure 5D). The expression of SDC4 was significantly higher in C1 than other clusters, and GPC3 was higher in C3 (Figure 5E,F). The validation cohort showed GPC3 was negatively correlated with all liver fibrosis markers ACTA2, COL1A1, COL1A2, COL3A1 and ductular reaction markers KRT7 and KRT19, while SDC4 was positively correlated with ductular reaction index KRT19 (Supplementary Figure S6).

### Hub HSPGs expression in BA liver tissues with different jaundice clearance standards

**Figure 6:**
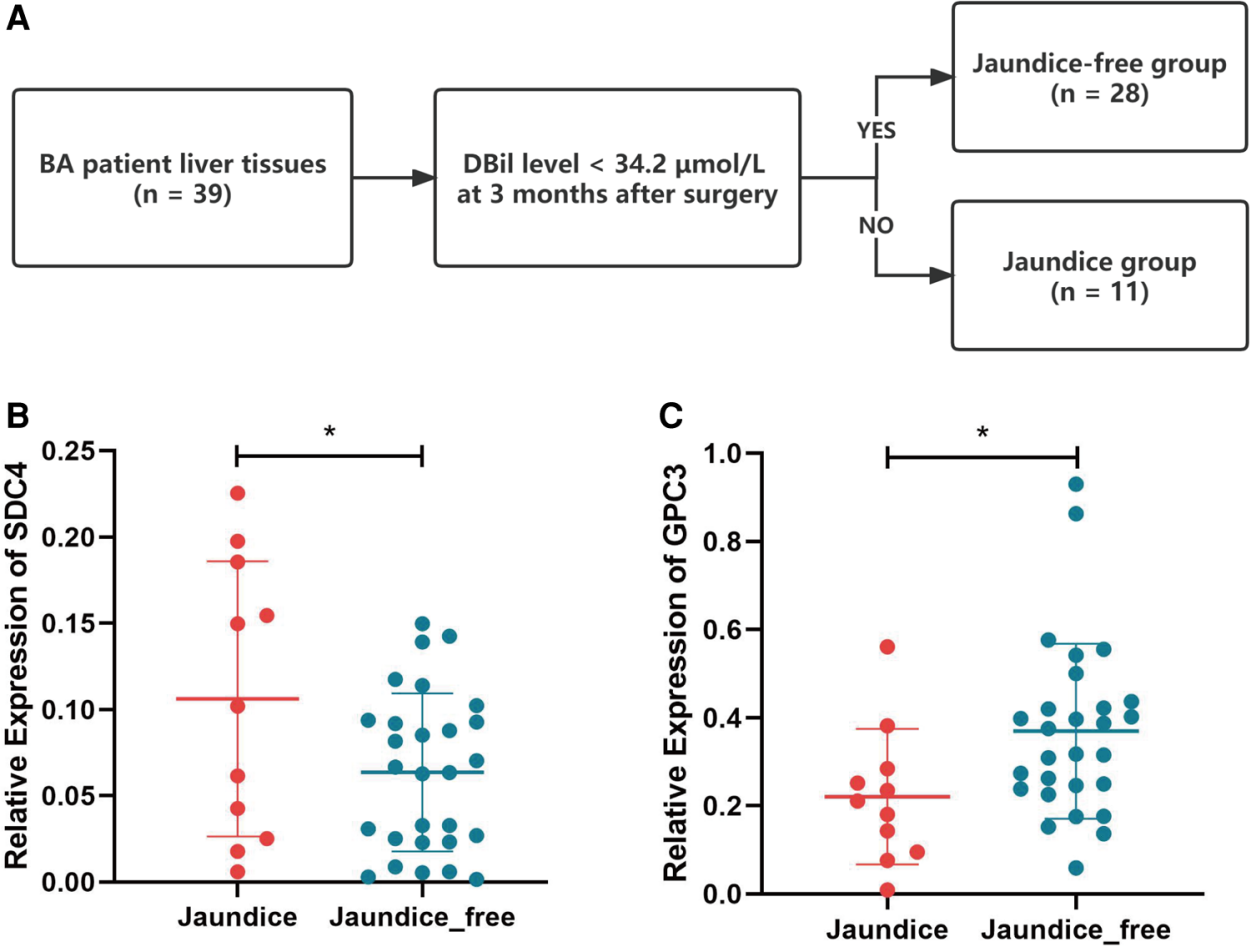
Hub HSPGs expression levels in patients with different jaundice clearance standards. (**A**) Patients were divided into 2 groups based on jaundice clearance standards. (**B**) GPC3 expression levels in 2 groups. (**C**) SDC4 expression levels in 2 groups. HSPG, heparan sulfate proteoglycan.

Based on jaundice clearance standard 3 months after Kasai surgery, 39 patients were divided into 2 groups, jaundice (*n* = 11) and jaundice-free (*n* = 28) (Figure 6A). The clinical characteristics of the patients in this study were similar in the 2 groups (Table 2). PCR results showed that SDC4 expression of liver tissues was significantly higher in jaundice group compared with jaundice-free group (Figure 6B). While GPC3 expression was significantly higher in jaundice-free group (Figure 6C).

**Table 2:** Patients’ information for SDC4 and GPC3 expression analysis.

	Jaundice	Jaundice-free	*p*-value
Age at Kasai (day)	55.09 ± 13.30	56.18 ± 16.95	0.81
Sex (male/female)	4/7	13/15	0.72
ALT level before surgery (U/L)	162.00 ± 90.85	195.88 ± 124.22	0.35
AST level before surgery (U/L)	253.50 ± 100.03	305.75 ± 246.31	0.59
Direct bilirubin level before surgery (μmol/L)	135.33 ± 37.16	143.74 ± 41.49	0.55

ALT, alanine aminotransferase; AST, aspartate aminotransferase.

### The expression of hub HSPGs with liver fibrosis and ductular reaction markers in BA liver tissues

**Figure 7:**
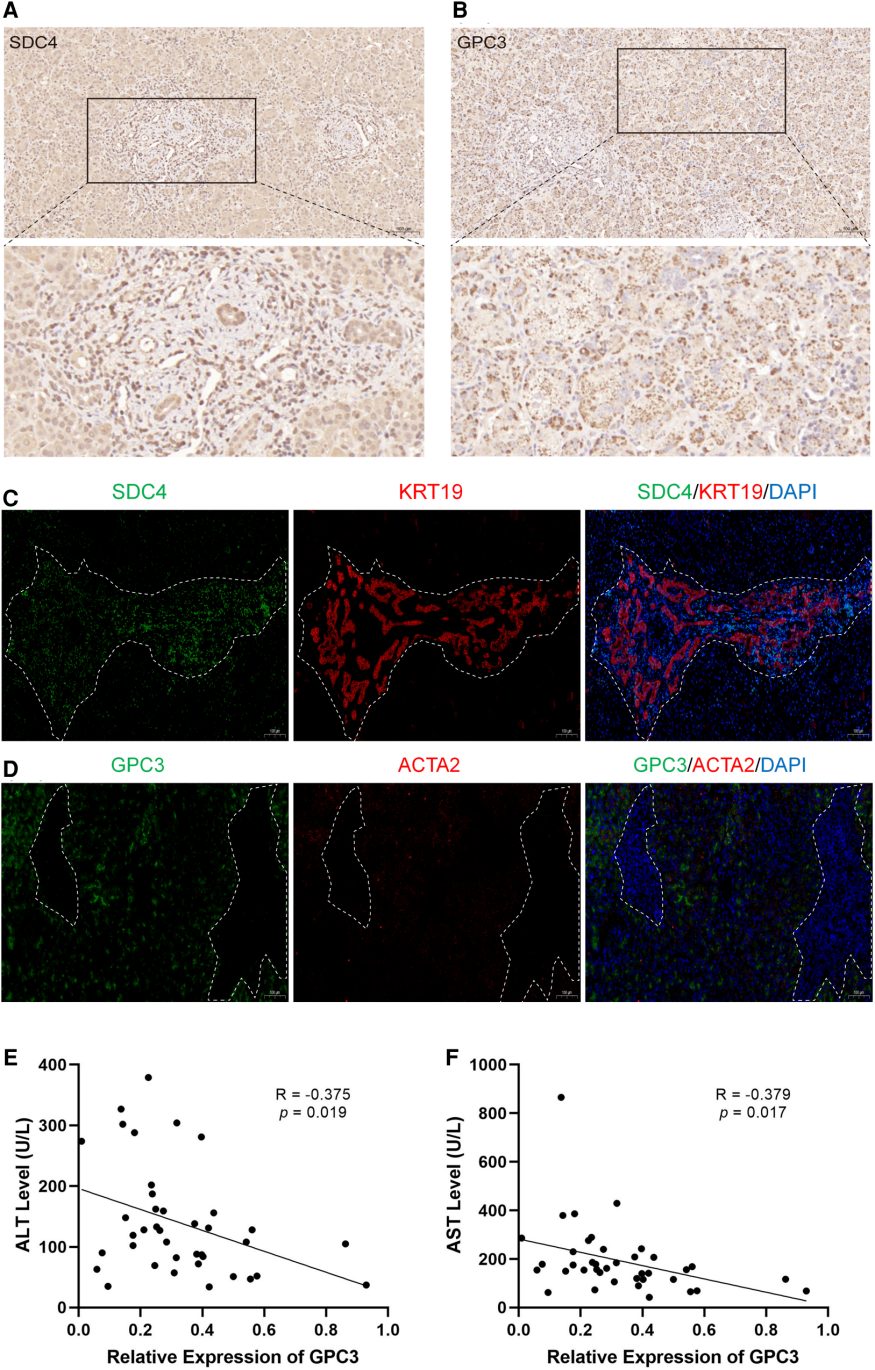
The expression of SDC4 and GPC3 with liver fibrosis and ductular reaction markers in BA liver tissues. Immunohistochemical staining of SDC4 (**A**) and GPC3 (**B**) in BA liver. Immunofluorescence of SDC4 and KRT19 (**C**), GPC3 and ACTA2 (**D**) in BA liver. The white dotted line marks out the extent of portal area. The correlation of relative expression of GPC3 with ALT (**E**) and AST (**F**). BA, biliary atresia; ALT, alanine aminotransferase; AST, aspartate aminotransferase.

To further validate the above observation, the expression of SDC4 and GPC3 were evaluated in BA liver tissue. SDC4 was strongly positive in portal areas and weakly positive in hepatocytes, while GPC3 had strong positivity in hepatocytes via immunohistochemical (IHC) staining (Figure 7A,B). Immunofluorescence assay showed the expression of SDC4 were mostly restricted with $KRT19^{+}$ cells (Figure 7C). GPC3, however, was high-expressed in hepatocytes rather than in portal area, which indicates it may have function in hepatocytes (Figure 7D). Finally, correlation analysis showed the expression levels of GPC3 in 39 BA patients have significantly negative correlation with alanine aminotransferase (ALT) and aspartate aminotransferase (AST) level (Figure 7E,F).

## Discussion

This study described the landscape of HSPGs in BA for the first time. We found that most HSPGs were high-expressed in BA liver tissue and positively associated with liver fibrosis, ductular reaction and immunocytes infiltration, indicating HSPGs may play important roles in these pathologic changes of BA.

Liver fibrosis is a key pathological feature of BA as an abnormal accumulation of extracellular matrix components. The extent of ductular reaction parallels mortality, and ductular reaction can be a therapeutic target to inhibit liver fibrosis and to promote liver regeneration (4). Immune system disorders, both innate and specific, are involved in BA (21). HSPGs have been widely reported to participate in these pathological changes in many diseases. Therefore, we would like to identify the exact role of HSPGs in BA and understand if there is a target to evaluate the disease stage.

When designing clinical trials based on the biological stages of BA, histopathology and molecular methods of staging liver disease may distinguish subgroups of patients at diagnosis (6). Disease classifications can be used to formulate individual management of BA patients after surgery and provide potential targets for personalized treatment. In this study, we found that BA can be classified into three main subtypes based on the expression of HSPGs. Whether different groups represented different hepatic pathological states was analyzed subsequently, which would provide potential sight for prognostic assessment.

Pathway enrichment analysis indicated that patients in the C1 subtype had more severe fibrosis and ductular reaction than in other subtypes. SDC4 was identified as the hub gene of this subtype. SDCs are transmembrane proteoglycans widely participating in the physical connection and cellular signaling transduction (22). SDC4 was reported participating in signal transduction and regulate cellular processes including proliferation, migration, adhesion (23). In hepatocellular carcinoma, SDC4 can interact with the actin cytoskeleton to facilitate the assembly of focal adhesions, ultimately leading to proliferation and metastasis of tumor cells (24). Moreover, the extracellular domain of SDC4 can be released into the serum and has been used as a potential biomarker for the diagnosis of non-alcoholic fatty liver disease (25). Interestingly, there was no difference in the expression level of SDC4 between BA and NC liver, while SDC4 was significantly higher in C1 than other subtypes within BA liver, indicating that SDC4 has the potential to predict prognosis rather than being used as marker for screening for BA. Our IHC and immunofluorescence results showed that SDC4 was expressed in portal areas, associated with the proliferation of bile ducts, indicating the high expression level of SDC4 in BA might be relative with the ductular reaction. Previous study also reported SDC4 was associated with ductular reaction in chornic cholestasis (10). Abnormal biliary duct proliferation is one of the important risk factors for the decreased native liver survival rate (4). Thus, we considered that SDC4 could be used as an indicator for poor prognosis in BA, because of relationship with the expansion of ductular cells.

Pathway enrichment of C3 subtype involved metabolic processes in the liver, indicating patients in the C3 subtype might have a relatively better liver condition. GPC3 was negatively correlated with all liver fibrosis and ductular reaction markers and subsequently selected as the hub gene for this subtype. In BA's liver tissues, we found that GPC3 was mostly expressed in hepatocytes, indicating GPC3 might stabilize the function of hepatocytes rather then participate in the liver fibrosis and ductular reaction directly. Further more, we found the expression level of GPC3 was negatively correlated with ALT and AST level, indicating that high expression level of GPC3 may reflect a relative slight liver injury. However, the specific function of GPC3 in BA pathological changes still need further investigation.

There are several limitations to this study. The molecular pathology of BA is complex and therefore other clinical or molecular indicators need to be combined to increase the sensitivity and specificity of molecular classification. Moreover, jaundice clearance is only one aspect of the prognosis, and larger cohort studies with longer follow-up are necessary.

## Conclusion

HSPGs are closely associated with liver fibrosis, ductular reaction and immunocytes infiltration in BA. A molecular classification of BA based on HSPGs may provide novel insights into personalized management of BA in the future.

## Data Availability

The datasets presented in this study can be found in online repositories. The names of the repository/repositories and accession number(s) can be found in the article/**Supplementary Material**.
