# Supplementary material for: Bioinformatics analysis identifies heparan sulfate proteoglycans acting as different progress subtypes of biliary atresia
Source: Front Pediatr. 2023 Feb 2;11:1065521. doi: 10.3389/fped.2023.1065521 (PMC9932896; doi:10.3389/fped.2023.1065521)
Supplement: Supplementary file 1 [file Datasheet1.pdf]

Supplementary Materials

Table S1. Transcriptome data of BA and NC liver tissues.

GEO	Platform	Tissue	Group	Samples (Number)			Author/Reference
				Total	BA	NC	
GSE122340	GPL16791	Liver	Discovery cohort	128	121	7	Luo Z[1]
			Validation cohort	50	50	0	

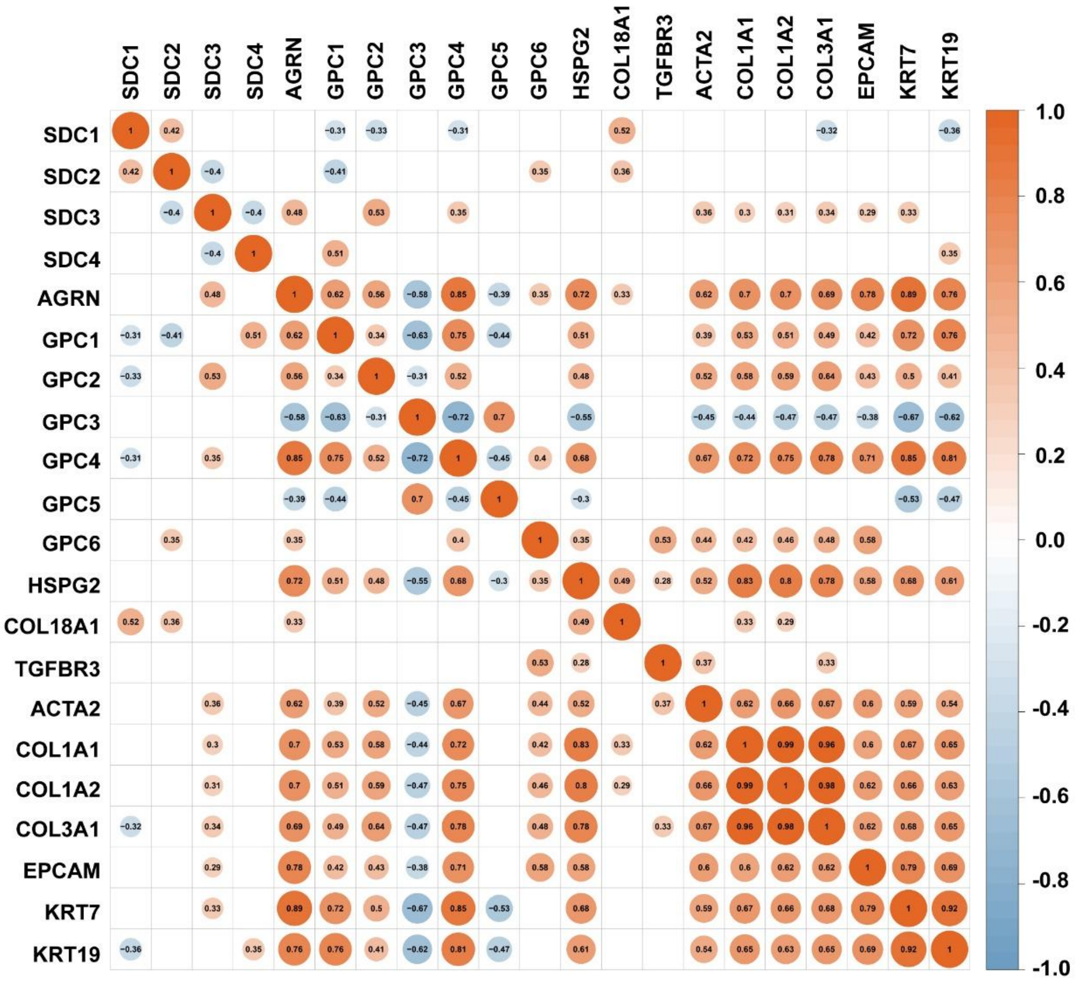

Figure S1. Correlation between heparan sulfate proteoglycans (HSPGs), liver fibrosis and ductular reaction of BA in validation cohort.

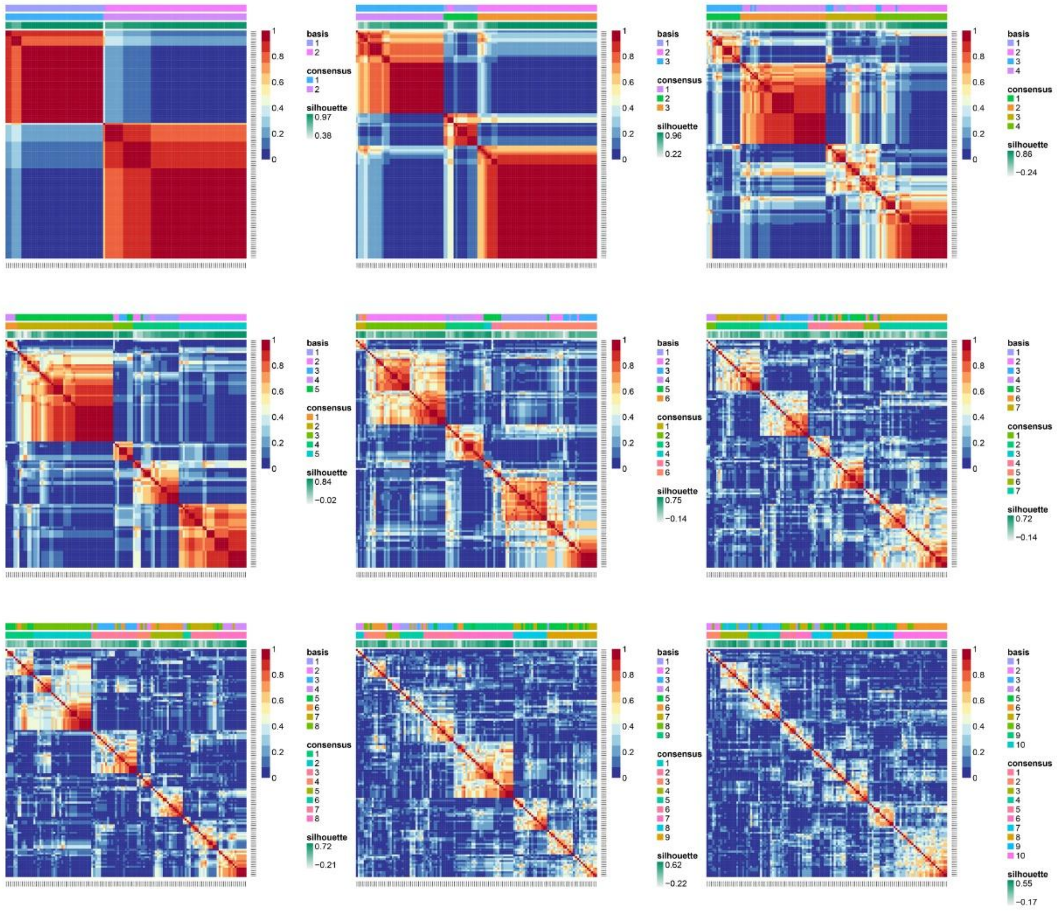

Figure S2. The consensus matrix heatmap for k = 2-10 was shown.

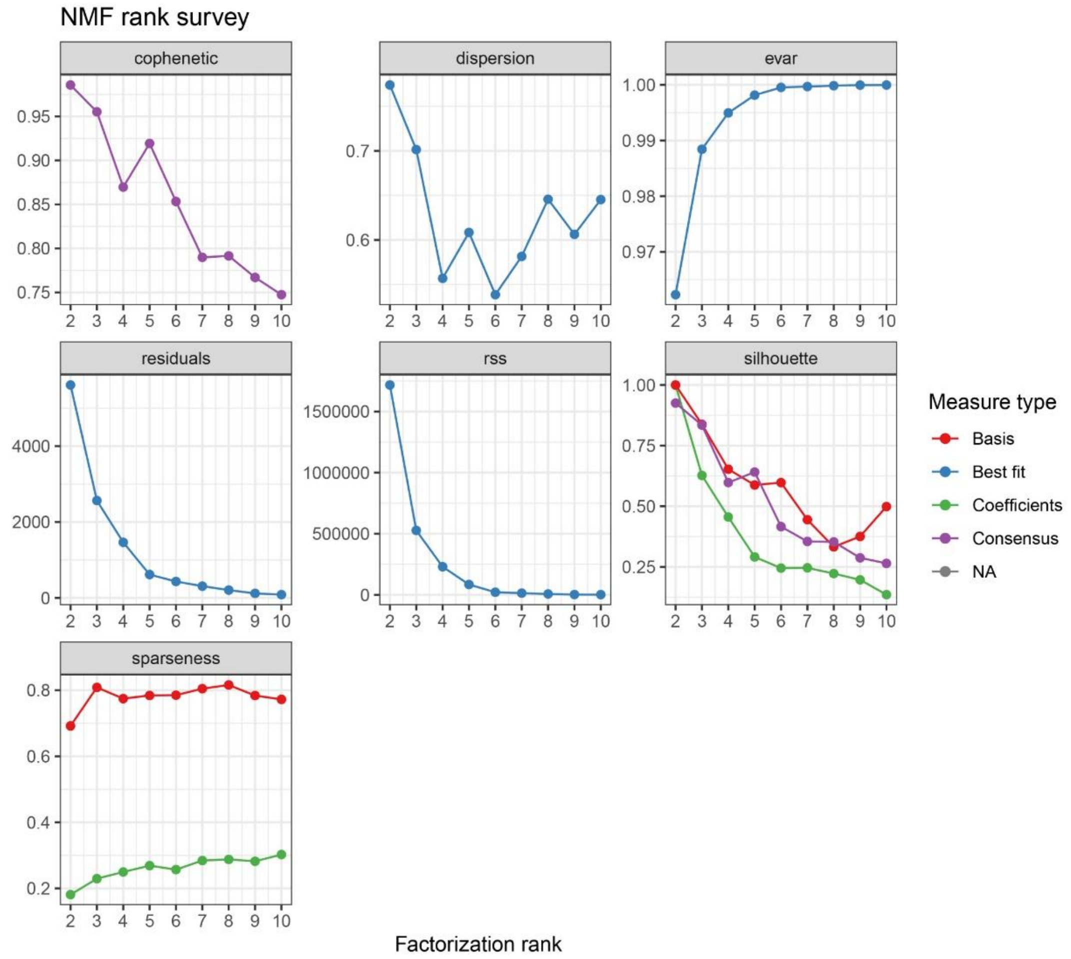

Figure S3. NMF rank survey for k = 2-10 was shown.

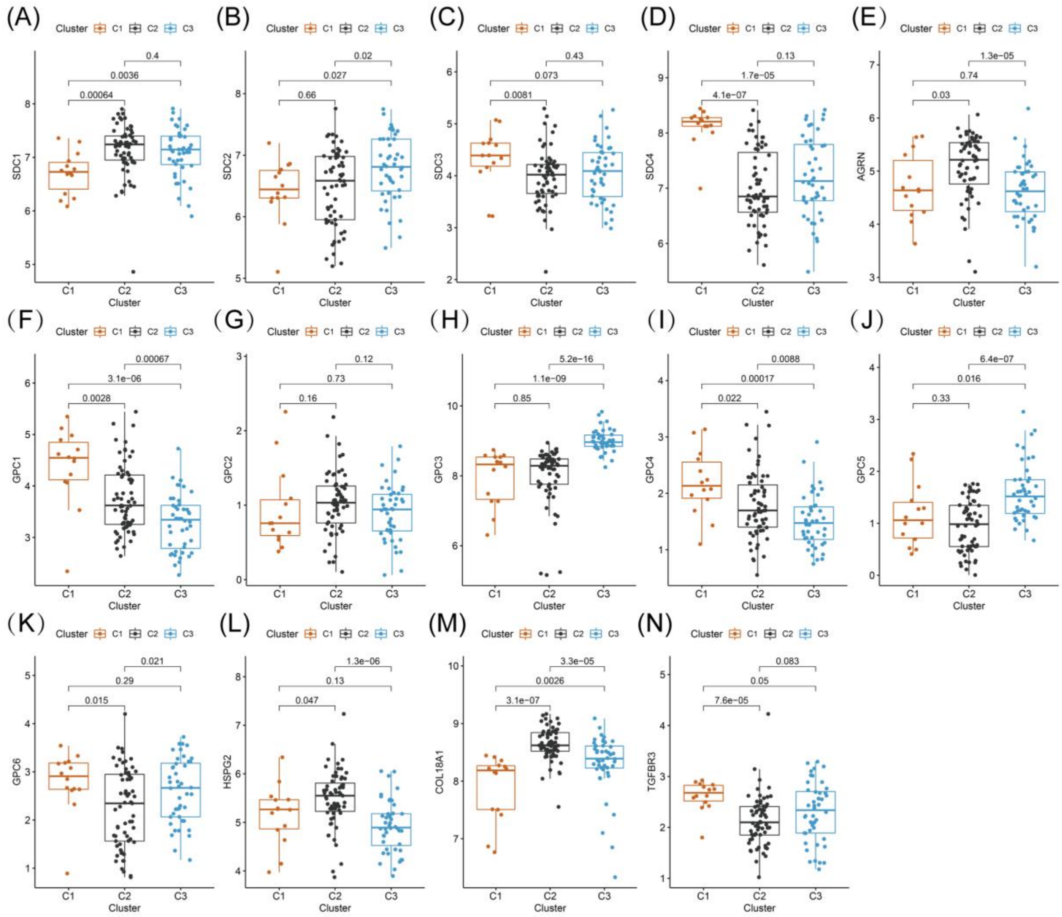

Figure S4. (A-N) The expression of Heparan sulfate proteoglycans (HSPGs) among three identified subtypes.

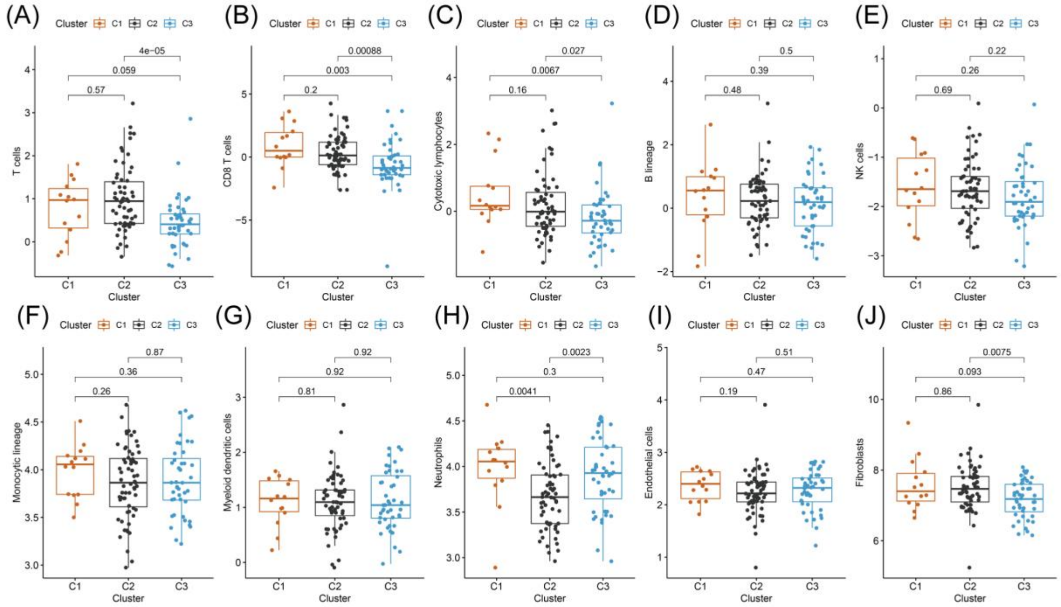

Figure S5. (A-J) The composition and abundance of immunocytes calculated by MCPcounter among three identified subtypes.

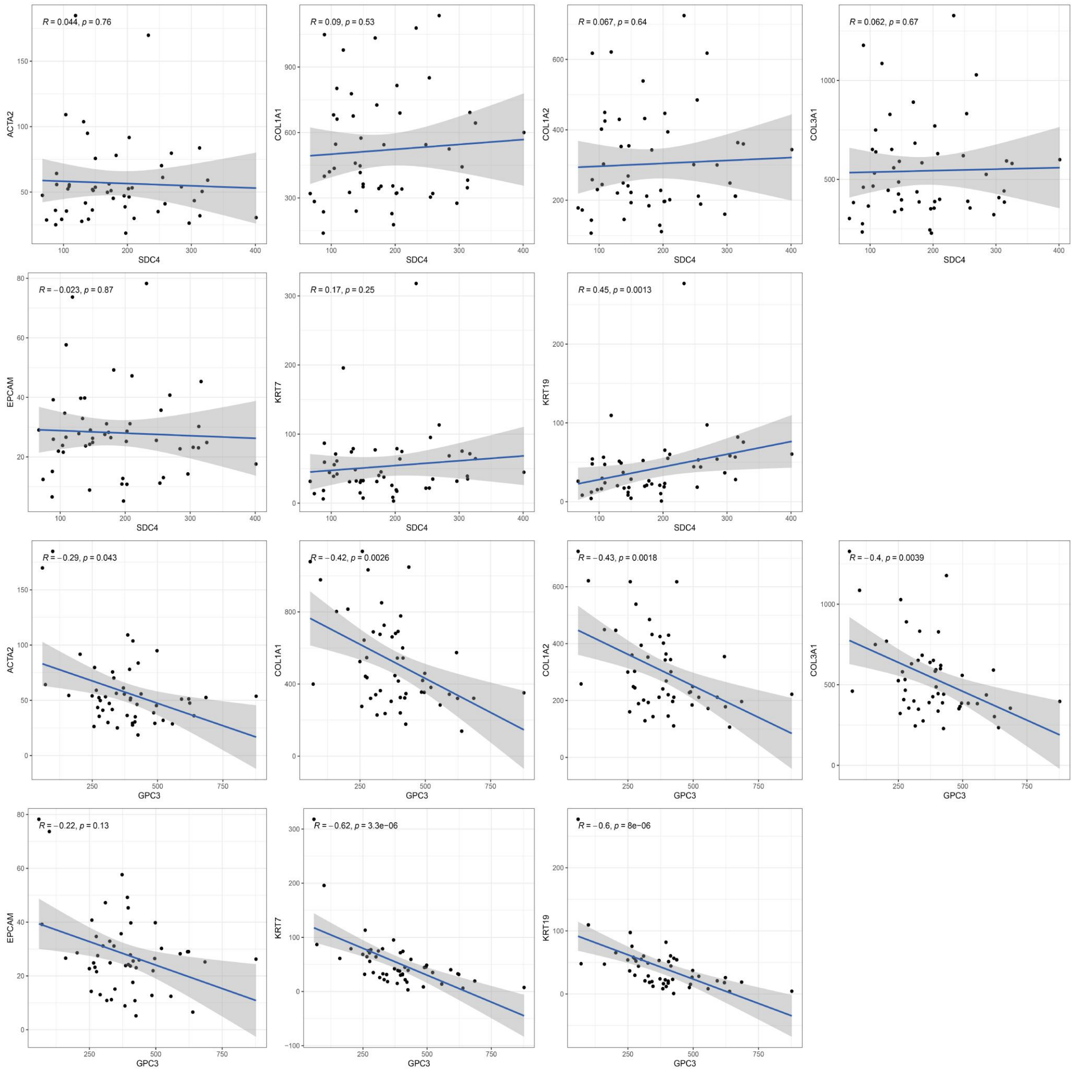

Figure S6. The correction analysis between the expression levels of hub HSPGs (SDC4 and GPC3) and fibrosis and ductular reaction signatures in the validation cohort.

References

[1] Z. Luo, P. Shivakumar, R. Mourya, S. Gutta, and J.A. Bezerra, Gene Expression Signatures Associated With Survival Times of Pediatric Patients With Biliary Atresia Identify Potential Therapeutic Agents. Gastroenterology 157 (2019) 1138-1152 e14.
